# Gamma entrainment as a functional target in deep brain stimulation

**DOI:** 10.4103/NRR.NRR-D-25-00510

**Published:** 2025-08-13

**Authors:** Bandy Chen

**Affiliations:** Department of Medicine, UC San Diego School of Medicine, La Jolla, CA, USA

Deep brain stimulation (DBS) is a neuromodulation tool that involves the delivery of electrical impulses to specific brain regions through implanted electrodes. The principle behind DBS is to modulate dysfunctional neural circuits without the need for permanent structural alterations to the brain. Initially developed as a treatment for movement disorders such as Parkinson’s disease (PD), DBS has expanded to encompass various neurological and psychiatric disorders. Until recently, DBS uses high-frequency electrical pulses to disrupt abnormal patterns of brain activity. Recent advances in neuroscience are shifting toward precision-based rhythm restoration with the goal of reinstating normal oscillatory patterns. Entrainment of brain activity within the gamma range (30–100 Hz), particularly around 40 Hz, has demonstrated potential in improving neurological disorders such as Alzheimer’s disease (AD) and PD (Deng et al., 2024). This represents a shift in the goal of DBS from silencing neural circuits to restoring physiological brain rhythms.

**Gamma oscillations as a biomarker for adaptive DBS:** Conventional DBS systems typically deliver continuous, open-loop stimulation at 130–185 Hz with the goal of suppressing pathological oscillations such as beta rhythms. The continuous stimulation changes the pattern of network firing and reduces synchronization of pathological circuits. It is applied regardless of the patient’s behavioral or physiological state, which can result in both therapeutic and unintended side effects. In contrast, adaptive DBS (aDBS) is a closed-loop system that uses real-time feedback from the patient’s brain activity to adjust stimulation parameters. Common biomarkers used in aDBS include beta oscillations and excessive beta-gamma coupling. However, the limited understanding of neural signatures associated with specific symptoms in neurological disorders treatable by DBS remains a major challenge. This is further complicated by technical difficulty of sensing brain signals during ongoing electrical stimulation and the lack of standardized algorithms to optimize feedback control. Defining neural biomarkers that remain measurable and trackable during stimulation at therapeutic amplitudes is essential.

Gamma oscillations, typically ranging from 30 to 100 Hz, are brain rhythms associated with cognition, perception, and movement. Narrow band gamma oscillations (30–80 Hz) are rhythmic and frequency-specific, reflecting synchronized network activity, while broadband gamma oscillations (70–150 Hz) are non-rhythmic and reflect asynchronous activity and general increases in local neural firing (Ichim et al., 2024). Broadband gamma activity in subcortical or cortical local field potentials is associated with initiation and execution of voluntary movements (Mathiopoulou et al., 2025). In PD patients, a narrower gamma frequency band in the range of 65–90 Hz, known as spontaneous finely tuned gamma (FTG), is associated with levodopa-induced dyskinesia (Olaru et al., 2024). This involves involuntary, often excessive movements as a side effect of chronic dopaminergic therapy. Levodopa-induced gamma oscillations closely track dyskinesia severity over time, showing similar temporal dynamics across the subthalamic nucleus and motor cortex. A proposed hypothesis is that repeated levodopa administration suppresses beta oscillations and induces cortical hyperexcitability, particularly in the primary motor cortex (Guttler et al., 2021). The shift from beta to high-frequency activity leads to the development of FTGs in the 65–90 Hz range. Although this presents as a pathological state, the established involvement of gamma-band activity in various motor processes raises important questions on how DBS modulates gamma oscillations.

DBS has been shown to entrain neural oscillations at subharmonic frequencies, most notably at one-half of the stimulation frequency (Sermon et al., 2023). For example, stimulation delivered at 80 Hz can induce gamma-band activity at approximately 40 Hz. It prompts further investigation into the functional significance of entrained gamma activity and whether such stimulation-induced rhythms contribute to motor control. Gamma-band activity (40–90 Hz) increases during voluntary movement and in response to effective dopaminergic medication, supporting its role as a prokinetic signal (Mathiopoulou et al., 2025). Within this context, an increase in prokinetic neuronal activity could enhance the responsiveness of the brain to stimulation and entrainment due to resonance with intrinsic network dynamics and lower threshold for phase-locking. The underlying framework suggests that if gamma activity reflects a movement-ready brain state, then DBS is more likely to achieve entrainment by locking onto and amplifying these beneficial oscillations. Entrained gamma activity (stimulation at 130 Hz with 1:2 gamma entrainment at 65 Hz) correlates with kinematic and clinical measures, with repetitive finger tapping enhancing or inducing gamma entrainment (Mathiopoulou et al., 2025). Patients with gamma entrainment demonstrate better motor performance, suggesting a functional prokinetic role for entrained gamma oscillations. There is some nuance to this observation, as some patients who displayed spontaneous FTG experience worsened dyskinesia with DBS (Oehrn et al., 2024). Patients without spontaneous FTG do not exhibit dyskinesia despite showing gamma entrainment and demonstrate better motor performance compared to those without entrained gamma (Oehrn et al., 2024). To conclude, dopaminergic medication broadly activates the motor network and makes the brain more prone to developing dyskinesia. In contrast, gamma activity induced by DBS activates that motor network in a more focused and controlled way that can improve movement but not enough to trigger dyskinesia. However, if the network is already in a highly active state due to medication, adding DBS-induced gamma entrainment could exceed the threshold and shift the network into a dyskinetic state. Consistent with this, DBS-induced gamma entrainment is less likely to occur when patients are in the OFF-dopaminergic medication state. These data indicate that the level of movement-related network activation, which is strongly influenced by dopamine, determines whether DBS-induced gamma entrainment will be beneficial or problematic. Reducing dopaminergic medication when using DBS is important to prevent overstimulation of the motor network. Future studies that account for the balance between prokinetic and antikinetic biomarkers may allow for optimal modulation of stimulation amplitude, sustaining therapeutic efficacy while minimizing the risk of dyskinesia.

**Figure 1:**
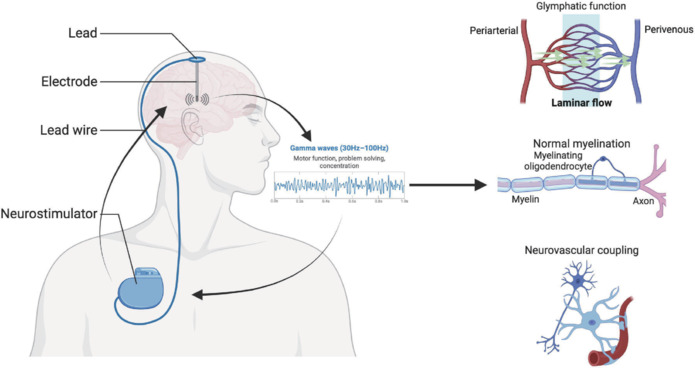
Bidirectional utility of gamma rhythms in closed-loop brain stimulation. Gamma rhythms serve as both a real-time biomarker for adaptive control and as a therapeutic target for neuromodulation. Fluctuations in gamma rhythms are associated with changes in behavioral state, cognitive demand, movement initiations, and transitions between sleep stages. This makes gamma oscillations a valuable feedback signal for closed-loop systems. Simultaneously, gamma entrainment has been shown to restore network synchrony, reduce neurotoxic molecules, and influence cognitive performance, motor control, emotional regulation, and sleep architecture. The mechanisms underlying gamma entrainment may involve enhanced glymphatic clearance, attenuation of demyelination, and improved neurovascular coupling, collectively supporting neuro-glial-vascular homeostasis and circuit integrity. Created with BioRender.com.

Along with dyskinesia, preliminary data suggest that gamma oscillations may also serve as a feedback signal for aDBS in the context of rapid eye movement (REM) sleep behavior disorder (RBD). Subthalamic gamma oscillations differ between phasic and tonic REM sleep and positively correlate with the severity of REM sleep fragmentation, with RBD individuals demonstrating stronger gamma activity during REM compared to non-RBD individuals (Guan et al., 2025). Simulations indicate that gamma-triggered feedback modulation more accurately tracks phasic REM activity than beta-triggered feedback modulation, supporting gamma oscillations as a candidate biomarker for aDBS in REM sleep disturbances (Guan et al., 2025). These sleep disturbances are both early indicators and modulators of disease progression across a range of neurological disorders. Targeting gamma oscillatory dynamics during sleep could represent a novel therapeutic strategy for improving cognitive and affective outcomes. 40 Hz visual stimulation during sleep reliably induces neuronal gamma activity at the stimulation frequency in different phases of sleep without compromising sleep quality (Hainke et al., 2025). Proof-of-principle for aDBS targeting N3 non-REM sleep has been demonstrated in PD patients, showing high specificity for stage N3 and good tolerability, with no observable adverse effects from stimulation adjustments (Smyth et al., 2023). These findings suggest the potential for gamma entrainment during specific sleep stages as a targeted intervention to enhance neurological outcomes. Overall, the widespread presence of gamma oscillations across diverse brain states and behavioral contexts underscores their utility as a feedback biomarker for neuromodulation. Given its ubiquity, gamma activity plays a fundamental role in maintaining neural homeostasis; consequently, restoring disrupted gamma dynamics observed in neurological disorders may offer therapeutic benefits (**Figure 1**).

**Therapeutic potential of DBS-induced gamma entrainment:** While gamma entrainment induced by DBS has been primarily explored in PD patients, abnormalities in gamma-band activity have been implicated across a broad spectrum in neurological and psychiatric disorders such as AD, where gamma deficits correlate with cognitive decline and amyloid pathology. Rodent studies demonstrate that gamma entrainment through DBS can modulate neural circuits involved in AD, particularly those related to memory and mood regulation. DBS-induced gamma entrainment of the ventral hippocampal CA1 region enhances the activity of parvalbumin-expressing interneurons and facilitates fear extinction (Lin et al., 2024). Furthermore, gamma entrainment via noninvasive transcranial alternating current stimulation of the lateral entorhinal cortex (EC) also induces parvalbumin-expressing interneuron activity in the CA1 region to modulate fear extinction (Lin et al., 2024). This indicates a top-down regulator motif, capable of driving low-gamma oscillations that enhance feedforward inhibition of “fear” neurons in the hippocampus to suppress conditioned fear responses. The finding provides proof of principle to selectively engage accessible top-down cortical circuits in a pathway-specific manner with precise cell type-specific modulations. While the lateral EC-ventral CA1 pathway is a critical circuit for fear extinction and mood regulation, it forms part of the broader EC-hippocampus axis, which is among the earliest affected pathways in AD. Extending DBS-induced gamma entrainment to encompass this broader axis offers potential applicability to other neurological or psychiatric conditions of similar phenomena and biomarkers by re-establishing functional connectivity and network synchrony. However, the translatability of gamma entrainment across different brain regions may require optimization as layer-specific stimulation of parvalbumin-positive cortical interneurons in mice has been shown to entrain brain rhythms at distinct frequencies depending on the cortical layer (David et al., 2023).

In addition to the EC-hippocampal circuit, the olfactory pathway is also selectively vulnerable in AD, with early pathological changes in the olfactory bulb. Suppression of olfactory bulb neurons or their projections to the piriform cortex reduces gamma oscillation power in limbic regions and induces depression-like behaviors (Li et al., 2023). Enhancing gamma oscillations from the olfactory bulb using closed-loop electrical neuromodulation ameliorates these behaviors. In contrast, delivering anti-phase closed-loop stimulation, which disrupts gamma synchrony, exacerbates depression-like behaviors. Interestingly, ketamine treatment restores limbic gamma power and reverses depression-like behaviors (Li et al., 2023). These findings establish a causal relationship between limbic gamma oscillations and depression-like behaviors, demonstrating that restoring these endogenous rhythms through direct stimulation or pharmacological treatment can alleviate depressive symptoms.

The amygdala plays a central control in emotional processing, social behavior, fear learning, and memory consolidation. Through its extensive connections with the EC, hippocampus, and olfactory regions, this dense anatomical connectivity places the amygdala within a network that is highly susceptible to early tau and amyloid-beta accumulation in AD. It is among the earliest limbic regions to undergo neurodegeneration, which contributes to neuropsychiatric symptoms and accelerates the deterioration of memory-related circuits. Prominent gamma oscillations are observed in the rat basolateral amygdala during the consolidation of contextual memory (Kanta et al., 2019). Modulating gamma activity through closed-loop signal processing and optogenetic stimulation during the consolidation phase can bidirectionally influence spatial memory strength. Because gamma oscillations manifest as brief, variable bursts, most modulation techniques lack the speed, specificity, and adaptability to target them effectively. The study demonstrates for the first time that utilizing a programmable signal processor that tracks gamma amplitude and phase in real time combined with precise phase-locked optogenetic stimulation, effectively overcomes these limitations (Kanta et al., 2019). This approach has been demonstrated in non-human primates using optogenetic stimulation with excitatory opsins, delivered via intracortical optrodes equipped with integrated light-emitting diodes (Zaaimi et al., 2023). A limitation of aDBS is that electrical stimulation generates artifacts that interfere with neural signal recording, therefore incorporation of optogenetics can enable artifact-free, high-fidelity modulation, and monitoring. Collectively, these findings highlight gamma entrainment as a promising therapeutic strategy across a range of neurological disorders. Future studies employing invasive gamma frequency stimulation, particularly through targeted, closed-loop approaches, may further enhance the efficacy of DBS.

**Conclusions:** Gamma oscillations hold dual promise as both a biomarker and a neuromodulatory target for DBS in neurological disorders. As a biomarker, gamma activity reflects dynamic brain states linked to movement, cognition, and affect, offering a physiological signal to guide real-time adjustments in stimulation. As a modulatory signal, gamma entrainment can be achieved through electrical, sensory, or optogenetic stimulation, and can restore neural circuits across various neurological disorders, including PD, AD, and depression. This dual functionality supports the integration of gamma oscillations into next-generation closed-loop neuromodulation systems aimed at enhancing both precision and clinical efficacy.

**Additional file:** *Open peer review report 1.*

OPEN PEER REVIEW REPORT 1
