# Supplementary material for: Gamma entrainment as a functional target in deep brain stimulation
Source: Neural Regen Res. 2025 Aug 13;21(7):2918–9. doi: 10.4103/NRR.NRR-D-25-00510 (PMC13378899; doi:10.4103/NRR.NRR-D-25-00510)
Supplement: Supplementary file 1 [file NRR-21-2918_Suppl1.pdf]

OPEN PEER REVIEW REPORT 1

Name of journal: Neural Regeneration Research
Manuscript NO: NRR-D-25-00510
Title: Gamma entrainment as a functional target in deep brain stimulation
Reviewer's Name: Luka Milosevic
Reviewer's country: Canada

COMMENTS TO AUTHORS

This is an interesting article about DBS-induced gamma entrainment. A few minor questions and comments are provided below.

"Entrainment of brain activity within the gamma range (30-100 Hz), particularly around 40 Hz, have shown significant potential in improving neurological disorders as Alzheimer's disease (AD) and PD." - Is this so? I think a reference is warranted.

"The paradigm shift from silencing neural circuits to restoring physiological brain rhythms promotes long-term network-level restoration." - maybe historically it was assumed that DBS silenced circuits, or it was hoped that DBS would produce said silencing, but it is highly unlikely the this is what DBS is/was actually doing (regardless of whether people intended for this to happen and/or thought that this was happening). Thus, maybe more careful and precise wording is required

"Common biomarkers used in aDBS include beta oscillations, local field potentials (LFPs), and phase-amplitude coupling" - safe to say that beta oscillations qualify as a biomarker. However, LFP is a general term for aggregate level oscillatory brain signals; which include beta oscillations; thus, the sentence is a bit redundant. Phase-amplitude coupling is again a rather general term / method; probably better to be more precise. Phase and amplitude of what and recorded where?

"while broadband gamma oscillations (70-150 Hz) are non-rhythmic and reflect asynchronous activity and general increases in local neural firing (Ichim et al., 2024). Broadband gamma activity, spanning 40-100 Hz," - two different definitions provided

"In PD patients, a narrower gamma frequency band in the range of 70-90 Hz, known as spontaneous finely tuned gamma (FTG), is associated with levodopainduced dyskinesia (Olaru et al., 2024)." - any ideas as to how / why it arises?

"Gamma-band activity increases during voluntary movement and in response to effective dopaminergic medication, supporting its role as a prokinetic signal (Mathiopoulou et al., 2025)." & "Entrained gamma activity correlates with kinematic and clinical measures, with repetitive finger tapping enhancing or inducing gamma entrainment (Mathiopoulou et al., 2025)."- in which range? Is this FTG or something else? Might be good to be specific about FTG vs general gamma (are these the same, different?).

"Within this context, an increase in prokinetic neuronal activity could enhance the brain's responsiveness to stimulation and entrainment" - how/why?

"The underlying framework suggests that if gamma activity reflects a movement-ready brain state, then DBS is more likely to achieve entrainment by locking onto and amplifying these beneficial

oscillations" - not sure DBS "locks on" to the rhythm. It's the other way around (given that DBS is entraining the rhythm, not the other way around)

"In contrast, gamma activity induced by DBS activates that motor network in a more focused and controlled way that can improve movement but not enough to trigger dyskinesia" - what does "enough" mean? My naïve guess is that DBS produces a stronger entrained gamma signal than dopaminergic medication (please correct me if wrong). If correct though, then not sure it would be appropriate to use the word "enough" since it likely surpasses. This would benefit from more precise wording.

I think that suggesting that there might be "Therapeutic potential of DBS-induced gamma entrainment" in AD and depression is quite far-reaching. I would caution against using insights from STN-DBS in PD and showing cortical gamma entrainment to speculate about other disorders, DBS targets, and circuits. For example, STN-DBS works for PD; but the same paradigm does not work for AD when applied in the fornix for example. I would use a more cautious word, eg. "Potential utility of similar phenomena/biomarkers in other disorders"

"Simultaneously, gamma entrainment has been shown to restore network synchrony, reduce neurotoxic molecules, and influence cognitive performance, motor control, emotional regulation, and sleep architecture. The mechanisms underlying gamma entrainment may involve enhanced glymphatic clearance, attenuation of demyelination, and improved neurovascular coupling, collectively supporting neuro-glial-vascular homeostasis and circuit integrity." - without appropriate references and elaboration, this feels very speculative and far reaching. Attenuation of demyelination or enhanced glymphatic clearance give rise to gamma entrainment? I'm not so sure. I would integrate such hypotheses into the main text with appropriate references, or remove. The claims, as provided currently in the figure legend, are unsubstantiated; and I wouldn't know where to look to convince myself of the plausibility of such hypotheses.
